# Effect of low-dose esketamine on cardio-biliary reflex and postoperative pain during laparoscopic cholecystectomy surgery: A randomized, controlled trail

**DOI:** 10.1371/journal.pone.0321892

**Published:** 2025-06-02

**Authors:** Xiaodong Zhang, Peng Duan, Yingjie Sun, Qi Na

**Affiliations:** 1 Department of Anesthesiology, Xiangyang Central Hospital, Affiliated Hospital of Hubei University of Arts and Science, Xiangyang, Hubei, China; 2 Department of Anesthesiology, General Hospital of Northern Theater Command, Shenyang, Liaoning, China; Monash University, ETHIOPIA

## Abstract

**Background:**

Cardio-biliary reflex can lead to cardiac arrest, brady-arrhythmia, cardiogenic shock, and other severe complications. NMDA receptor antagonists have been shown to have the effect of anti-vagal reflex. However, the regulation of vagus reflex by esketamine, an NMDA receptor antagonist, remains unclear. Our study aims to investigate intravenous low-dose esketamine on cardio-biliary reflex.

**Methods:**

In this randomized controlled trial, adult patients undergoing laparoscopic cholecystectomy were allocated in a 1:1 ratio to esketamine group or control group. 5 minutes before surgical incision, participants in the esketamine group received 0.3 mg/kg of esketamine, while the control group received an equivalent volume of normal saline. The primary outcome was the occurrence of cardio-biliary reflex. Postoperative pain was assessed using the Visual Analogue Scale (VAS) on days 1, 2, and 3 post-surgery.

**Results:**

Our final analysis included 140 participants. The incidence of the cardio-biliary reflex occurred in 15 patients (21.4%) in the control group compared with 6 patients (8.6%) in the esketamine group (relative risk 0.34; 95%confidence interval (95% CI): 0.125–0.947; *P *< 0.05). Patients in the esketamine group reported lower pain intensity with movement on postoperative days (POD)1, 2, and 3 with mean differences (MD) of 0.59, 0.70, and 0.47 points respectively (all *P* < 0.05). Additionally, pain intensity at rest was also lower in the esketamine group at all observation time points (POD1: MD 0.51, POD2: MD 0.40, POD3: MD 0.30, all *P* < 0.05).

**Conclusions:**

Therapeutic use of low-dose esketamine significantly reduces the occurrence of cardio-biliary reflex and postoperative pain in patients undergoing laparoscopic cholecystectomy.

## Introduction

Laparoscopic cholecystectomy (LC) surgery has emerged as the gold standard for treating symptomatic cholelithiasis, acute cholecystitis, and other gallbladder diseases. In the US, gallbladder disease impacts approximately 20 million individuals, significantly increasing familial economic burdens [1,2]. After laparoscopic surgery, some complications are still unavoidable. Commonly reported issues include nausea, vomiting, biliary reflex, abdominal pain, and surgical infection [3,4]. Of particular concern is the cardio-biliary reflex, a prevalent vagus nerve reflex that can lead to cardiac arrest, arrhythmia, cardiogenic shock, and other severe complications. The vagus reflex is mediated by the trigeminal nerve (afferent limb) and the vagus nerve (efferent limb). During LC, manipulation of the gallbladder can activate the vagus nerve, triggering adverse cardiac events such as sinus bradycardia, arrhythmia, and asystole. Therefore, devising effective strategies to prevent cardio-biliary reflex is crucial for the safety of both surgery and anesthesia.

Another significant challenge following LC surgery is post-operative pain, which causes anxiety, depression, insomnia, and can prolong hospital stays, inflate medical costs, and hinder early postoperative recovery [5]. Inadequate pain management post-LC can increase risk of developing persistent post-surgery pain [6,7]. While opioids are commonly used for post-surgical pain management, their adverse complications-tolerance, addiction, nausea, vomiting, respiratory depression, and hyperalgesia-necessitate alternatives. Non-opioid analgesics are increasingly recognized as vital components of multimodal analgesia reducing opioid-related side effects [8]. Esketamine, a potent N-methyl-D-aspartate (NMDA) receptor antagonist, has shown a promise in reducing pain intensity and opioid requirements post-surgery [9]. NMDA receptors modulate spinal neuronal activity, influence wind-up and central sensitization of dorsal horn sensory neurons, and are crucial in the development of pain states [10]. Vagus nerve excitation increases gallbladder motility and its effect can be abolished by administering NMDA recptors [10]. Esketamine is the dextral form of ketamine and has a higher affinity for NMDA recptor. Our study aims to investigate whether intravenous low-dose esketamine administered before skin incision can effectively reduce the incidence of cardio-biliary reflex and alleviate postoperative pain in patients undergoing LC.

## Methods

This randomized, double-blind, placebo-controlled trial was conducted at Xiangyang Central Hospital, Affiliated Hospital of Hubei University of Arts and Science, Xiangyang, China, from January 15, 2024, to April 15, 2024. The study protocol received approval from the Hospital Research Ethics Committee (Ethical approval No.2023-113-02) and conformed to the Declaration of Helsinki and CONSORT guidelines. The trial was registered at the Chinese Clinical Trial Registry (January 02, 2024; ChiCTR2400079362; https://www.chictr.org.cn/). Written or verbal consent from all participants.

### Inclusion and exclusion criteria

A total of 148 patients aged 18–60 years, and who were scheduled for elective LC with American Society of Anesthesiologists (ASA) grade I to III was enrolled to participate in this trial. Exclusion criteria included: patients were contraindication to esketamine (e.g., allergic to esketamine and any schizophrenia, mania, and any other mental illness); pathological sinus node syndrome; heart rate < 45 beats/min; mental, language or communication barrier; converted to open surgery; contraindications to anesthetic drugs (remifentanil, sufentanil, propofol). Patients participating in another study within 4 weeks before enrollment were also excluded.

### Randomization and double-blind

A biostatistician uses a computer-generated list of random numbers in a 1:1 ratio. Each participant is assigned a unique random number; participants are then ranked in ascending or descending order based on these random numbers. According to pre-established sample sizes for each treatment group, subjects are selected sequentially by their random number to be allocated to the respective treatment arms. Based on the random seeds, patients were allocated into two groups: esketamine group (0.3 mg/kg of esketamine, Jiangsu hengrui Pharmaceutical Co., Ltd.) or control group (same volume of normal saline). The randomization sequence was sealed in numbered opaque envelopes managed by a research coordinator, who was not involved in anesthesia management, perioperative care, or postoperative follow-up. During the study period, the research coordinator open envelope before anesthesia induction and prepared the study drugs according to group assignment. The study drugs stored the drugs with identical appearance sterile syringes produced by the same manufacturer. Blinding was maintained for patients, anesthesiologists, surgeons, and investigators. Furthermore, all surgeries were performed by same surgeries and anesthesiologists’ team.

### Anaesthesia, perioperative and intraoperative care

Preoperative anesthesia evaluation occurred one day before surgery. Standard fasting guidelines were followed. After patients enter to the operating room, electrocardiogram, heart rate, peripheral pulse oxygen saturation, non-invasive blood pressure, and nasopharyngeal temperature was routinely monitored. Ringer lactate (5 mL/kg) infusion via venous access was achieved by peripheral intravenous catheter. The participants routinely inhaled oxygen at 3 L/min through a nasal catheter. General anaesthesia was induced with sufentanil (0.4 µg/kg), etomidate (0.2 mg/kg), rocuronium (0.9 mg/kg). Endotracheal intubation was performed by the same anesthetist, and mechanical ventilation was used to maintain PetCO2 in the range of 35–45 mmHg. Awake fiberoptic bronchoscopy tracheal intubation was performed for those subjects with predicted difficult airway. After tracheal intubation, an anesthesia nurse took trail drugs from trial coordinator and intravenous administration of it. Anesthesia was maintained with propofol (60–120 µg/kg/min), remifentanil (0.2–0.5 µg/kg/min), and sevoflurane (1–2%). Anaesthesia depth was targeted to maintain BIS between 40 and 60 by increasing or decreasing intravenous propofol, or sufentail. Intraoperative noninvasive mean blood pressure was maintained within 20% of the baseline values and above 60 mmHg, which was achieved by intravenous infusion of phenylephrine or nitroglycerin (20 µg increments or continuous infusion). Severe bradycardia was defined as intraoperative patient’s heart rate < 45 beats/ min, and 0.25 mg atropine was administrated by peripheral intravenous catheter. After surgery, all of the subjects were transferred to the post-anaesthesia care unit (PACU) for recovery before returning to the ward. After the surgery, patients were administered intravenous flurbiprofen axetil 50mg, and a bilateral transversus abdominis plane block was performed under ultrasound guidance, with each side receiving 20mL of 0.375% ropivacaine.

### Data collection and measurements

The baseline clinical characteristics of all the patients were collected: age, weight, height, smoking habits, drinking, allergies, chronic pain, marital status, education, history of disease, ASA status. Non-invasive blood pressure, heart rate, respiratory rate and pulse oxygen saturation was recorded at baseline (T0), immediately before esketamine or normal saline injection (T1), 5 minutes after esketamine or normal saline injection (T2), immediately before pulling the gallbladder (T3), 5min after pulling the gallbladder (T4), immediately after end of operation (T5), immediately upon awakening (T6). Various intraoperative parameters noted included: duration of surgery and anaesthesia, mechanical ventilation time, extubation time, infusion volume, estimated blood loss.

The primary outcome was the incidence of cardio-biliary reflex which is defined by a decrease in heart rate and blood pressure by greater than 20% following traction of the gallbladder. The cardio-biliary reflex also mimics the special ECG changes of acute coronary syndrome, such as ST-segment elevation and T-wave inversion. The reflex most commonly results in sinus bradycardia. Furthermore, it also has a reported association with reduced arterial pressure, arrhythmia, asystole, and even cardiac arrest. Atropine or epinephrine was used to maintain heart rate within 20% of its baseline in each time.

Mini-mental state examination (MMSE) and hospital anxiety and depression scale (HAD) assess patient’s anxiety, depression and cognitive function before and 1 day after surgery [11]. Post-operative pain intensity at rest and during movement was evaluated with visual analogue scale (VAS) pain score on postoperative day 1, 2, and 3 [12]. If the patient’s pain score was more than 3 points or requiring analgesic drugs, a low-dose (2–3ug/kg) sufentanil would be given. In addition, the number of patients who requested extra rescue pain treatment during the postoperative analgesia was also recorded. Adverse events including nausea, vomiting, dizziness, increased salivation, delirium, drowsiness, and pruritus were recorded after recovery from anesthesia and on the first day after surgery.

### Statistical analysis

The sample size calculation used PASS 11.0 software (NCSS, LLC. Kaysville, Utah). According to our preliminary observations, the incidence of cardio-biliary reflex in control group was about 27%. Sample size calculation was based on an anticipated r incidence of 6% in the esketamine group, with a 2-sided alpha error of 0.05 and 90% power. Considering a 20% dropout rate, a total of 148 subjects were planned for enrollment. Data are expressed as mean ± SD, median(inter-quartile range) or absolute numbers. Categorical variables were analysed with χ^2^ tests, continuity correction χ^2^ tests, or Fisher’s exact tests; between-group differences were expressed as relative risks (RR) and 95% CI. Continuous variables with normal distribution were analyzed with independent-sample t-tests and Linear Mixed-Effects Models; between-group differences were expressed as mean difference (MD) and 95% CI. Those with non-normal were analyzed with Manne-Whitney U tests. 2-way ANOVA with Bonferroni post hoc test was employed for pain and intraoperative data comparisons. Statistical analyses were using the GraphPad Prism (GraphPad Prism 8.3.0, San Diego, CA) and SPSS 25.0 (IBM, Armonk, NY, USA).

## Result

### Baseline characteristics

**Fig 1:**
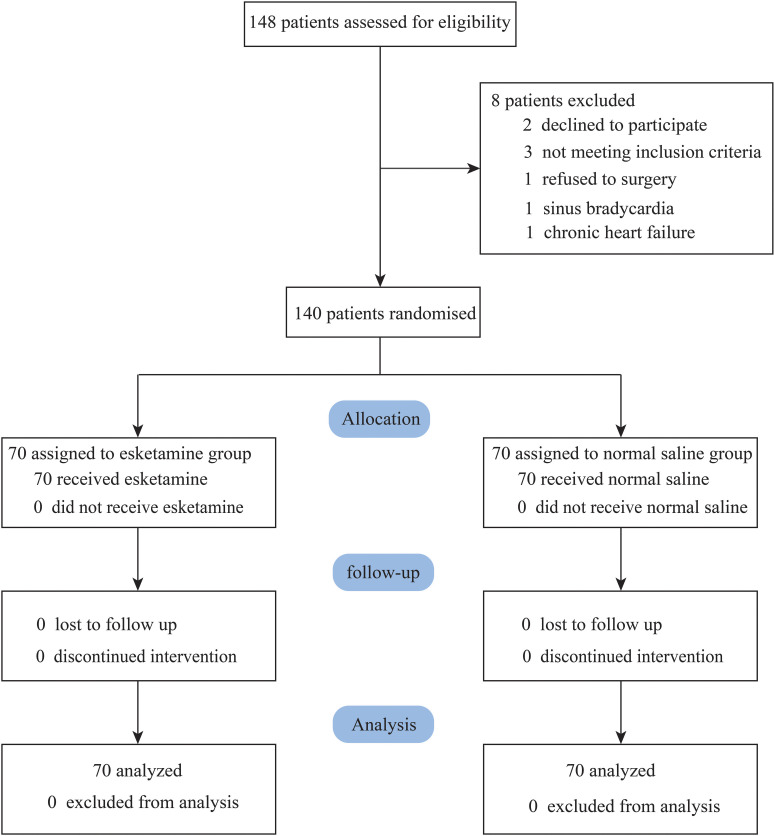
Flow chart.

148 patients were initially assessed for participation in our study. Finally,140 eligible patients were randomized to esketamine group (n = 70) or control group (n = 70, Fig 1). 8 patients were excluded from this study: 3 did not satisfy the inclusion criteria, 2 patients declined to participate our study, 1 patient refused surgery, 1 patient was excluded due to sinus bradycardia, 1 patient was excluded on account of chronic heart failure (Fig 1). A comparative analysis of demographic and clinical characteristics exhibited no significant difference across two groups (*P* > 0.05, Table 1).

**Table 1:** Baseline data of all patients.

	Control group (n = 70)	Esketamine group (n = 70)	*P* value
Age(years)	46.4 ± 13.7	47.5 ± 15.8	0.661
Height(cm)	166.67 ± 7.428	168.41 ± 7.794	0.178
Weight(kg)	66.8 ± 11.9	70.8 ± 15.6	0.086
Gender			0.123
Male	36 (51.4%)	45 (64.3%)	
Female	34 (48.6%)	25 (35.7%)	
ASA status			0.050
I	14 (20%)	22 (31.4%)	
II	56 (80)	45 (64.3%)	
III	0	3 (4.3%)	
Drinking	23 (32.9%)	29 (41.4%)	0.294
Smoking	24 (34.3%)	18 (25.8%)	0.471
Marital status			0.820
Single	11 (15.7%)	12 (17.1%)	
Married	59 (84.3%)	58 (82.9%)	
Level of education			0.293
Primary school	5 (7.1%)	5 (7.1%)	
Junior school	17 (24.3%)	21 (30%)	
Senior school	24 (34.3%)	14 (20%)	
University	24 (34.3%)	30 (42.9%)	
Allergic for esketamine	0	0	NA
History of surgery	7 (10%)	14 (20%)	0.098
History of disease			
Consciousness or mental disease	0	0	NA
Heart disease	3 (4.3%)	3 (4.3%)	> 0.05
Pulmonary disease	1 (1.4%)	2 (2.9%)	> 0.05
Liver disease	1 (1.4%)	0	> 0.05
Neurological diseases	0	2 (2.9%)	0.496
Hypertension	6 (8.6%)	5 (7.1%)	0.753
Hyperlipidemia	1 (1.4%)	3 (4.3%)	0.310
Diabetes mellitus	4 (5.7%)	5 (7.1%)	0.730
History of chronic pain	0	0	NA
Body temperature	36.4 (36, 36.5)	36.5 (36, 36.5)	0.146

Data are presented as mean ± SD, median (inter-quartile range), or number (%).

### Intraoperative medical indicators

**Fig 2:**
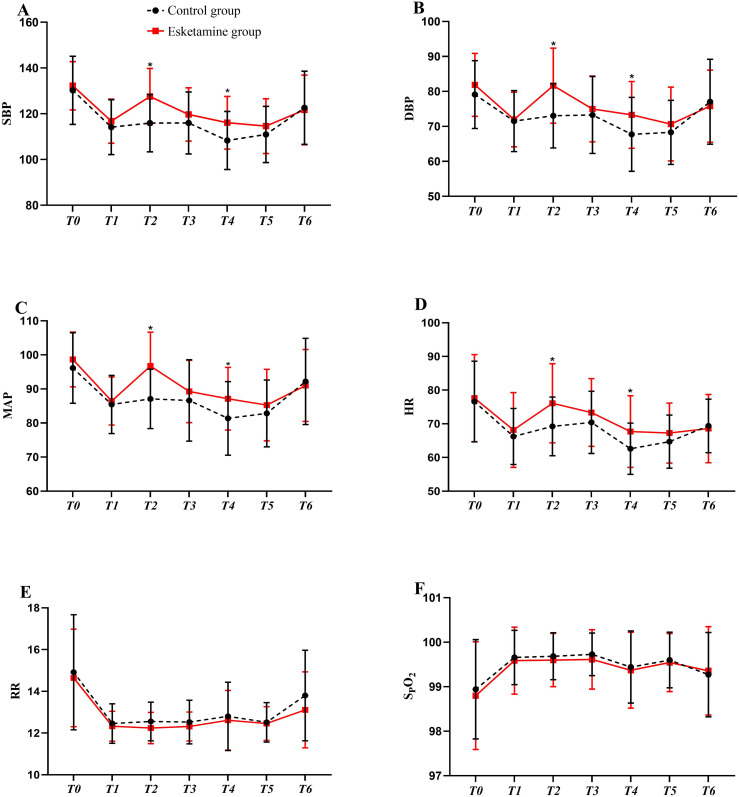
Comparison of hemodynamic and oxygen saturation parameters between groups. Note: T0: baseline; T1: immediately before esketamine or normal saline injection; T2:5 minutes after esketamine or normal saline injection; T3: immediately before pulling the gallbladder; T4: 5min after pulling the gallbladder; T5: immediately after end of operation; T6: immediately upon awakening. *P < 0.05 vs. the control group.

Intraoperative administration esketamine decreased in the incidence of gallbladder-heart reflexes compared to the control group [control group vs. esketamine group; 21.4% (15/70) vs 8.6% (6/70), relative risk (RR) 0.34; 95% confidence interval (95% CI): 0.125–0.947; *P *< 0.033]. Intraoperative parameters, including duration of anaesthesia and surgery, the times of mechanical ventilation, extubation and recovery, infusion volume, and estimated blood loss, were well balanced across groups (*P* > 0.05, Table 2). Blood pressure values and heart beats were significantly higher in the esketamine group at 5 minutes after esketamine injection and 5min after pulling the gallbladder (*P* < 0.05, Fig 2A, B, C, D). At other monitored timepoints, there were no significant differences in blood pressure and heart rate between the two groups (*P *> 0.05, Fig 2A, B, C, D). At any time point during the operation, no statistical difference was observed in the fluctuation of respiratory rate and pulse oxygen saturation between the 2 groups (*P *> 0.05, Fig 2E, F).

**Table 2:** Intraoperative data.

	Control group (n = 70)	Esketamine group (n = 70)	*P* value
Cardio-biliary reflex	15 (21.4%)	6 (8.6%)	0.033
Duration of anaesthesia (min)	107.20 ± 52.131	100.99 ± 43.983	0.447
Mechanical ventilation times (min)	123.57 ± 52.580	118.76 ± 46.457	0.566
Duration of surgery (min)	73.20 ± 45.043	63.86 ± 38.210	0.188
Extubation times (min)	3.06 ± 3.387	3.07 ± 2.975	0.979
Recovery times (min)	13.36 ± 9.545	14.70 ± 12.970	0.487
Infusion volume (mL)	744.29 ± 281.500	726.43 ± 252.053	0.693
Estimated blood loss (mL)	20 (10,30)	20 (10,30)	0.993

Results are presented as mean ± SD, median(inter-quartile range) or number (%).

### Comparison of postoperative VAS score

**Fig 3:**
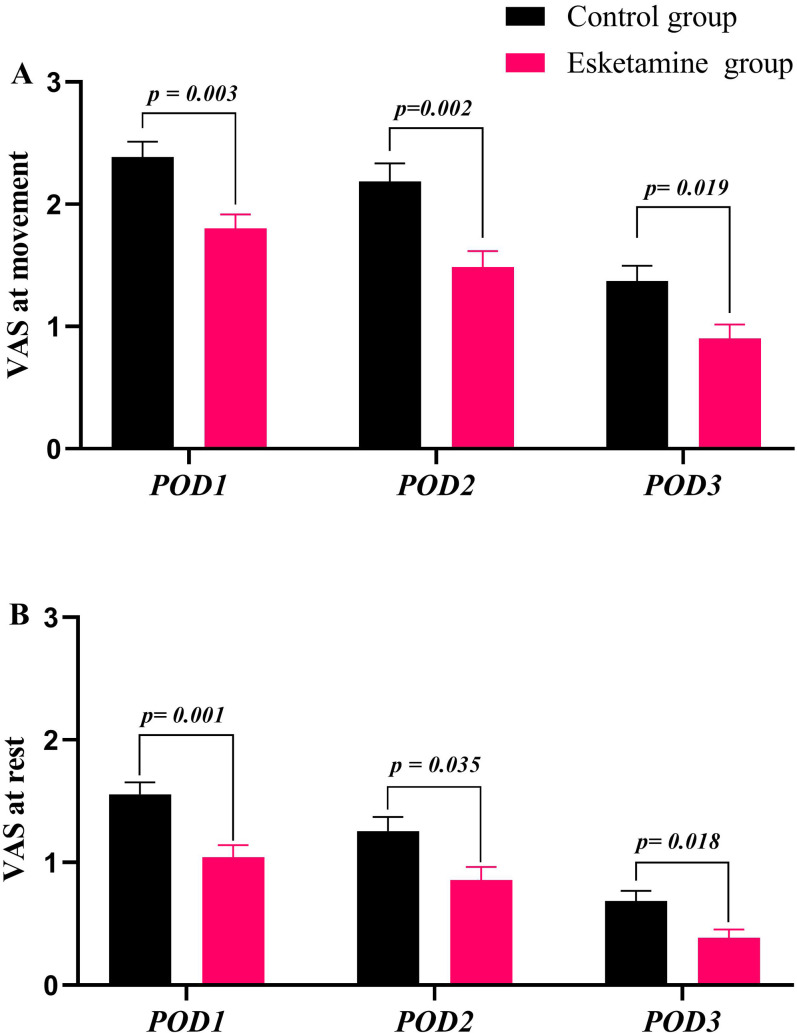
Pain VAS scores. Note: POD1: postoperative day 1; POD2: postoperative day 2; POD3: postoperative day 3.

Better analgesic effect was achieved in esketamine group rather than that in the control group. Patients in the esketamine group experienced a significantly lower pain intensity with movement compared to the control group on postoperative days 1, 2, and 3 [i.e., POD 1: control group vs. esketamine group, mean difference (MD) 0.59 points, 95% CI 0.17–1.0; POD 2: MD 0.70 points, 95% CI 0.22–1.18; POD 3: MD 0.47 points, 95% CI 0.06–0.88); all *P *< 0.05, Fig 3A]. At observation time points, pain intensity at rest was lower in the esketamine group than in the control [i.e., POD 1: control group vs. esketamine group, MD 0.51 points, 95% CI 0.18–0.85; POD 2: MD 0.40 points, 95% CI 0.02–0.78; POD3: MD 0.30 points, 95% CI 0.04–0.56; all *P *< 0.05, Fig 3B].

### Comparison of H3AD and MMSE score

The Preoperative HAD and MMSE score did not differ between the two groups (*P* > 0.05, Table 3). The HAD-A and HAD-D score was lower in the esketamine group compared to the control group at POD1 (*P* < 0.05, Table 3). The differences were no clinically significant, as all the patients’ scores were lower than seven points, indicating that the patients were free of anxiety and depression. There was no significant difference in MMSE between the two groups at POD1 (*P* > 0.05, Table 3).

**Table 3:** Perioperative HAD and MMSE scores.

	Control group (n = 70)	Esketamine group (n = 70)	*P* value
Preoperative-HAD-A	0 (0,1)	0 (0,1)	0.546
POD1-HAD- A	0 (0,2)	0 (0,1)	0.010*
Preoperative-HAD-D	0 (0,1)	0 (0,0)	0.549
POD1-HAD-D	0 (0,2)	0 (0,0.25)	0.006*
Preoperative-MMSE	30 (29,30)	30 (29,30)	0.179
POD1-MMSE	30 (29,30)	30 (30,30)	0.052

Results are presented as median (inter-quartile range). POD1: postoperative day 1. *P < 0.05 vs. the control group.

### Comparison of complications and analgesics requirements between two groups

There were no statistically significant differences in the incidence of nausea, vomiting, dizziness, recovery time, glandular secretion rate, and the number of patients requiring analgesia between the two groups (*P* > 0.05, Table 4). Both groups demonstrated similar frequencies of drowsiness and pruritus, with no significant differences observed (*P* > 0.05, Table 4). Importantly, there were no reported cases of psychiatric symptoms in any of the patients during the study period. Fewer subjects in the esketamine group asked for supplemental analgesics than in the control group [17.1% (12/70) vs. 7.1% (5/70); relative risk 1.50, 95% CI 1.05–2.14, *P* *<* 0.05, Table 4].

**Table 4:** Comparison of adverse events.

	Control group (n = 70)	Esketamine group (n = 70)	*P* value
Adverse events on recovery period			
Nausea and vomiting	5 (7.1%)	3 (4.3%)	0.466
Dizzy	4 (5.7%)	8 (11.4%)	0.237
Increased gland secretion	0	3 (4.3%)	0.080
Adverse events on post-surgery day 1			
Nausea and vomiting	7 (10%)	8 (11.4%)	0.785
Drowsiness	5 (7.1%)	6 (8.6%)	0.753
Pruritus	2 (2.9%)	1 (1.4%)	> 0.999
Dizzy	9 (12.9%)	11 (15.7%)	0.629
Delirium	0	0	NA
Illusion	0	0	NA
Number of patients requiring analgesics	12 (17.1%)	5 (7.1%)	0.070

Data are expressed as median (inter-quartile range) or absolute numbers.

## Discussion

In this study, we evaluated the effects of intraoperative single low-dose esketamine on cardio-biliary reflex and postoperative pain. Results demonstrated that low-dose esketamine significantly reduced the occurrence of cardio-biliary reflex and post-surgery pain and the requirement for supplemental analgesics within 72 h, without increasing adverse events, including nausea, vomiting, dizzy, psychiatric symptoms and others.

The cardio-biliary reflex, characterized by reflex sinus bradycardia and other electrocardiogram changes during acute cholecystitis or biliary colic, has been a major concern in such surgeries. In 1971, O’Reilly and Krauthamer firstly described the development of reflex sinus bradycardia and other various electrocardiogram changes in the setting of acute cholecystitis or biliary colic, known as Cope’s sign or the cardio-biliary reflex [13,14]. This reflex is incited by acute cholecystitis pain of the gallbladder, which leads to increased activation of autonomic neurons in the reflex arc inducing heart-related adverse events [15,16]. During LC, the surgeon manipulates the gallbladder can activate the vagus nerve inducing cardio-biliary reflex. Previous studies have demonstrated that presynaptic N-methyl-D-aspartate (NMDA) receptors expressed on vagal afferent terminals are involved in regulating vagal afferent activity [17]. Our study showed that a single dose of esketamine could effectively reducing the incidence of this reflex. This aligns with prior research highlighting the role of NMDA receptors in regulating vagal afferent activity [17]. Nonselective NMDA receptor antagonists inhibit vagus signaling by modulating various ion channels [17]. Ketamine has been shown to inhibit vagus-induced oculocardiac reflexes by excitating the sympathetic nerve [18]. Esketamine may inhibit vagal reflexes by the same mechanism. The specific reason may be that esketamine’s ability to deepen anesthesia and modulate vagal responses could be key to mitigating this reflex. The specific mechanism by which esketamine inhibits vagal reflex during cholecystectomy needs to be confirmed by further animal or clinical studies.

Small doses of esketamine has been shown to be beneficial in maintaining hemodynamic stability during LC [19]. In our study, we observed that blood pressure values and heart beats were higher in the esketamine group than that in the normal saline group at 5 minutes after esketamine injection and 5min after pulling the gallbladder. Currently, multicenter, double-blind randomized clinical trial of 903 women who were administered 0.25 mg/kg esketamine or saline before incision showed that the mean arterial pressure and heart rate were higher in the esketamine group than in the control group at 5 minutes after study drug administration [20]. Esketamine may stimulate the sympathetic nervous system to increase blood pressure and heart rate, especially when given in high doses [21]. In Zhang et al. ‘s study, a single intravenous injection of 0.15 mg/kg of esketamine was associated with a reduced risk of hypotension during cesarean section under spinal anesthesia with ropivacaine at 12 mg [22]. The reason for this may be due to esketamine exerts sympathetic excitatory effect that counteracted the cardiovascular inhibitory effect of anesthetic drugs, adverse reflexes [23]. The sympathetic excitatory effect of esketamine is related to two aspects:1) Esketamine blocks the sodium channel of brain stem parasympathetic neurons and inhibits parasympathetic nerve activity; 2) Esketamine inhibits NO release and enhances sympathetic nerve activity [24].

Severe acute postoperative pain often occurs in patients undergoing abdominal surgery [25]. Laparoscopic cholecystectomy is a minimally invasive procedure, but many patients still complain of moderate to severe pain-surgery [26]. Although non-opioid medications and regional/neuraxial techniques have been propose to improve analgesia, postoperative analgesia remains suboptimal [27]. Previous studies have shown that esketamine is effective in reducing pain intensity in the rest and movement in the short period after surgery [28]. In our study, intravenous administration of esketamine 5 minutes before the surgical incision effectively improved postoperative pain. Ktamine (bolus 0.2 mg/kg over 30 min followed by 0.12 mg/ kg/h for 24 h)) decreases pain intensity and opioid requirements following spinal fusion surgery in opioid-tolerant patients [29]. In addition, A recent meta-analysis of 30 randomized controlled trials, including 1,865 patients undergoing elective spinal surgery, showed that perioperative low doses of ketamine reduced pain intensity and opioid consumption at 12, 24, and 48 hours, with no increase in adverse events [30]. In this study, low doses of esketamine reduced post-operative static and dynamic pain 72 h postoperatively. Clinically significant difference was also observed in reducing the requirement for opioids. Esketamine is an NMDA receptor inhibitor, which can inhibit hyperalgesia and prolong postoperative analgesia [31,32]. Prior research has shown that preemptive low-doses ketamine can provide an adequate postoperative analgesia and increases the analgesic effect of tramadol following laparoscopic cholecystectomy [33].

Anxiety and depression often occur in surgical patients, which has become a worldwide public health problem [34]. Due to patients’ worries about the results of surgery, patients usually experience serious anxiety and depression. Effective measures to prevent anxiety and depression during perioperative period are conducive to rapid recovery after surgery. The FDA updated the approval of intranasal esketamine can improve adults with major depression and suicidal ideation and behavior [35]. In addition, the results of a systematic review and data analysis of 2,903 patients indicate that ketamine and esketamine are effective and safe for the treatment of patients with depression [36]. Low doses of ketamine and esketamine have been reported to be effective in alleviating postpartum depression [22,37]. However, in our results showed that low-doses of esketamine statistically reduced HAD scores, but our study found no significant clinical difference in these aspects, possibly due to the young patient demographic and small sample size.

Low-dose ketamine infusion is recommended to improve postoperative analgesia [38]. However, low-dose ketamine still produce side-effects, including psychiatric symptoms [39]. However, our findings confirm that the use of low doses esketamine in patients undergoing laparoscopic cholecystectomy is safe and does not increase the risk of psychiatric symptoms. A single intravenous injection of esketamine increased glandular secretion in patients undergoing thyroidectomy, and their results were consistent with the findings of this study [40]. No significant difference was found in the incidence of nausea, vomiting, dizzy, drowsiness and other complications in our study. The side effects of low doses of esketamine need to be further evaluated in large, multi-center studies.

## Limitation

Firstly, the relatively small sample size may not fully underpowered to detect the side effects of esketamine. Second, we only tested the effects of a fixed dose of esketamine (0.3 mg/kg). Fourth, more comprehensive investigations into biomarkers of the cardio-biliary reflex and rapid ECG changes associated with this reflex are warranted. Thirdly, we included a small number of elderly patients, and did not conduct subgroup analysis by age, so it is necessary to further explore the occurrence of outcomes at different ages.

## Conclusion

In conclusion, intraoperative low-dose esketamine effectively reduces the occurrence of cardio-biliary reflex and postoperative pain in patients undergoing laparoscopic cholecystectomy. Moreover, it stabilizes intraoperative circulation without increasing adverse complications. The current findings are only confirmed the effectiveness of intravenous low-dose esketamine of Chinese population.

## Supporting information

S1 File Clinical trial protocol. (DOCX)

S2 File CONSORT checklist. (DOCX)
