# Supplementary material for: Effect of low-dose esketamine on cardio-biliary reflex and postoperative pain during laparoscopic cholecystectomy surgery: A randomized, controlled trail
Source: PLoS One. 2025 Jun 2;20(5):e0321892. doi: 10.1371/journal.pone.0321892 (PMC12129155; doi:10.1371/journal.pone.0321892)
Supplement: S1 File — (DOCX) [file pone.0321892.s001.docx]

**Effect of low-dose esketamine on biliary-cardiac reflex and postoperative pain during laparoscopic cholecystectomy surgery: a randomized, controlled study**

**1. Background**

Laparoscopic cholecystectomy is widely used in clinical practice due to its minimal trauma and rapid postoperative recovery. The gallbladder and biliary tract are areas densely innervated by the vagus nerve. During surgery, stimulation, traction, and dissection of the gallbladder or exploration of the common bile duct can cause biliary-cardiac reflex, leading to reflex coronary artery spasm, myocardial ischemia, cardiac function suppression, bradycardia, arrhythmia, and in severe cases, cardiac arrest. Previous literature reports that the incidence of circulatory arrest during biliary surgery is 3.07 times that of non-biliary surgery, with an occurrence rate of biliary-cardiac reflex ranging from 30% to 75%. Therefore, exploring an appropriate anesthesia management strategy to prevent biliary-cardiac reflex is crucial for the prognosis of patients undergoing laparoscopic cholecystectomy. Administering a low dose of esketamine after anesthesia induction, utilizing its sympathetic stimulation effect to raise blood pressure, increase heart rate, and suppress adverse reflexes, may help counteract vagal reflexes during cholecystectomy. Esketamine is the right-handed isomer of ketamine, exhibiting similar pharmacological characteristics by blocking NMDA receptors for analgesic effects. It has twice the affinity for NMDA receptors as racemic ketamine, with no difference in onset and duration of action but higher bioavailability and shorter half-life. In the nervous system, NMDA receptors are involved in pain signal transmission and amplification. Inflammatory factors released from tissue injury or stimulation increase NMDA receptor activity, leading to pain signal transmission and amplification. Hence, inhibiting NMDA receptor activity can alleviate pain perception. Therefore, we investigated the effect of low-dose esketamine on biliary-cardiac reflex during cholecystectomy and postoperative pain.

- 1. **Primary Objective**

To observe the effect of administering low-dose esketamine after anesthesia induction on biliary-cardiac reflex and postoperative pain during cholecystectomy.

- 1. **Secondary Objectives**

1. The incidence of adverse reactions to perioperative use of esketamine.
2. The effects of perioperative esketamine use on postoperative depression, anxiety, and cognitive function in patients.

**3. Study Design**

**3.1 Study Nature**

This is a prospective, single-center, double-blind, randomized controlled trial assessing the impact of esketamine on biliary-cardiac reflex and postoperative pain in patients scheduled for elective laparoscopic cholecystectomy.

**3.2 Sample Size Calculation**

Based on pre-experiment and literature reports, it is estimated that the incidence of biliary-cardiac reflex during surgery is 40% in the saline group. Assuming a 25% reduction in the incidence of biliary-cardiac reflex in the esketamine group, setting α=0.05 for a two-sided test and β=10%, PASS software calculations require 124 patients in total, equally divided into experimental and control groups. Considering a potential dropout rate of 20%, a total of 148 patients will be recruited.

**3.3 Randomization and Blinding**

Patients will be randomized into two groups: control group C and experimental group K using the Research Randomizer website. The results will be stored in opaque envelopes, managed by a researcher not involved in patient recruitment, data collection, perioperative management, or postoperative follow-up. Before surgery, the coordinator will prepare the drugs according to the randomization results and provide them to the anesthesiologist responsible, labeled as "research drug" with the patient's random number. The anesthesiologists, surgeons, nurses, and follow-up personnel involved in the trial will be blinded to the group assignments until the end of the trial when all data have been recorded and analyzed.

**Emergency Unblinding:** In case of emergencies requiring knowledge of the treatment received, the principal investigator can decide to open the envelope to reveal the group assignment. Once opened, the case will be considered a dropout, and the reason recorded. The patient will not be included in efficacy analysis but will be included in adverse reaction analysis. If more than 20% of the envelopes are opened, the double-blind trial will be invalidated.

**3.4 Drug Intervention**

Group K: 0.3 mg/kg esketamine administered before skin incision.

Group C: Equivalent dose of saline administered before skin incision.

**4. Study Subjects**

**4.1** **Inclusion Criteria:**

ASA classification I-III.

Age ≥18 years, weight 50-100 kg, gender not restricted.

Scheduled for elective cholecystectomy under general anesthesia.

Willing to sign informed consent.

**4. 2 Exclusion Criteria**

Known allergy to esketamine hydrochloride or its excipients.

Severe cardiovascular, respiratory, hematologic, endocrine, or hepatic/renal dysfunction.

Risk of elevated blood pressure, intraocular pressure (e.g., glaucoma), or intracranial pressure.

Neurological or psychiatric disorders.

Alcohol abuse or long-term use of sedatives or analgesics.

Surgery or use of study drugs within one week before the trial.

Participation in other drug clinical trials within the last three months.

Communication barriers (e.g., reading/writing difficulties, language barrier, visual/hearing impairment) hindering study completion.

Refusal to sign informed consent.

4.3 Withdrawal Criteria

Occurrence of serious adverse events.

Protocol violations (e.g., unexpected ICU transfer, poor patient compliance).

Patient request to withdraw.

Loss to follow-up with no efficacy data collected.

5.1 Study Flow

Obtain written informed consent from patients before surgery, screen according to inclusion/exclusion criteria, and randomize using the randomization website. Store the randomization numbers in opaque envelopes. Preoperatively, introduce patients to the use of the Visual Analog Scale (VAS) for pain, the Hospital Anxiety and Depression Scale (HAD), and the Mini-Mental State Examination (MMSE).

All patients will undergo general anesthesia with intubation using a video laryngoscope, and be monitored using a Chinese multifunctional monitor for invasive arterial pressure, heart rate, SpO2, temperature, and end-tidal CO2. Anesthesia will be induced with propofol (2-3 mg/kg), rocuronium (0.6-1 mg/kg), and sufentanil (0.25-0.5 µg/kg). After loss of consciousness (absence of corneal and eyelid reflexes), tracheal intubation will be performed. Anesthesia will be maintained with remifentanil (0.1-0.3 µg/kg/min), propofol (3-4 mg/kg/h), and sevoflurane (0.7-1.0 MAC), with dosage adjusted by the anesthesiologist.

Definition of Biliary-Cardiac Reflex: During gallbladder manipulation or traction, if heart rate decreases (HR < 60 bpm) or drops by ≥20% from baseline along with a ≥20% drop in blood pressure from baseline, it will be classified as a biliary-cardiac reflex. If reflex occurs, surgery will be paused, and 0.5 mg of atropine will be administered intravenously. If recovery is not achieved quickly, the operation will be stopped, and rescue medications such as ephedrine, isoproterenol, or epinephrine will be administered.

Surgery will be performed by the same team of surgeons. Sevoflurane will be stopped 30 minutes before the expected end of surgery, and propofol and remifentanil infusions will be stopped at the end of the surgery. Patients will be observed for half an hour in the operating room after waking and restoration of spontaneous breathing before being transferred to the ward.

Postoperative Rescue Analgesia: Sufentanil will be titrated for analgesia. If VAS score > 3 (moderate to severe pain) or upon patient request, rescue analgesia will be administered and recorded. If VAS score ≤ 3 or if there is no request, no additional analgesia will be provided.

**5.2 Follow-up Plan**

Screening Period: 0-7 days before surgery, screen according to inclusion/exclusion criteria. Provide informed consent and complete the following assessments:

1. Basic information: name (initials), age, height, weight, medical history, preoperative diagnosis, ASA classification, comorbidities, marital status.
2. Preoperative depression/anxiety (HAD scale) and cognitive function (MMSE), pain assessment (VAS).
3. Laboratory tests: blood routine, biochemistry, urine routine, coagulation function within one week before consent.
4. Other auxiliary examinations: ECG, cardiac function.
5. All medications used within three days before surgery.

**5.2.** Follow-up Period

Evaluation Items Include:

Vital Signs upon Room Entry: Record the patient's vital signs upon entry into the operating room.

Surgery and Anesthesia Records: Record blood pressure, heart rate, oxygen saturation (SpO2), surgical duration, blood loss, fluid infusion, urine output before and after drug administration, anesthesia duration, and incidence of biliary-cardiac reflex.

Intraoperative Unexpected Events: Record any decrease in oxygen saturation (<90%), hypotension, hypertension, bradycardia, tachycardia, laryngospasm, intraoperative awareness, post-extubation nausea and vomiting, salivation, increased secretions, dizziness, restlessness, agitation, cognitive impairment, disorientation, respiratory depression, diplopia, blurred vision, hallucinations, and nightmares.

Postoperative Pain Assessment: Evaluate the intensity of postoperative pain at 24, 48, and 72 hours using the Visual Analog Scale (VAS) in both resting and movement states (e.g., coughing or deep breathing).

Usage of Postoperative Rescue Analgesics: Record the time of the first request for rescue analgesics and the total amount of rescue analgesics used within 72 hours.

Postoperative Depression/Anxiety and Cognitive Function: Assess using the Hospital Anxiety and Depression Scale (HAD) and the Mini-Mental State Examination (MMSE) at postoperative follow-ups.

Adverse Reactions: Record all adverse reactions within 72 hours postoperatively, including dizziness, nausea, vomiting, subjective reports of dreams, skin itching, hallucinations, diplopia, postoperative delirium, agitation, and other mental-related side effects.

**6.1 Study Drugs**

Esketamine Hydrochloride Injection: Main ingredient: esketamine hydrochloride. Chemical name: (S)-2-(2-chlorophenyl)-2-(methylamino) cyclohexanone hydrochloride. Manufacturer: Jiangsu Hengrui Medicine Co., Ltd. Specification: 2 ml: 50 mg. Approval number: National Drug Approval H20193336.

6.2 Equipment Used

Monitor: BeneVision N15 multifunctional monitor by Shenzhen Mindray Bio-Medical Electronics Co., Ltd., China. Registration certificate number: National Medical Device Registration Certificate 20173210926.
